# Ethics of instantaneous contact tracing using mobile phone apps in the control of the COVID-19 pandemic

**DOI:** 10.1136/medethics-2020-106314

**Published:** 2020-05-04

**Authors:** Michael J Parker, Christophe Fraser, Lucie Abeler-Dörner, David Bonsall

**Affiliations:** 1 Wellcome Centre for Ethics and the Humanities and Ethox Centre, The Ethox Centre, Nuffield Department of Population Health, University of Oxford, Oxford, UK; 2 Big Data Institute, University of Oxford, Oxford, UK; 3 Wellcome Centre for Human Genomics, University of Oxford, Oxford, UK; 4 Oxford University NHS Trust, University of Oxford, Oxford, UK

**Keywords:** ethics

## Abstract

In this paper we discuss ethical implications of the use of mobile phone apps in the control of the COVID-19 pandemic. Contact tracing is a well-established feature of public health practice during infectious disease outbreaks and epidemics. However, the high proportion of pre-symptomatic transmission in COVID-19 means that standard contact tracing methods are too slow to stop the progression of infection through the population. To address this problem, many countries around the world have deployed or are developing mobile phone apps capable of supporting instantaneous contact tracing. Informed by the on-going mapping of ‘proximity events’ these apps are intended both to inform public health policy and to provide alerts to individuals who have been in contact with a person with the infection. The proposed use of mobile phone data for ‘intelligent physical distancing’ in such contexts raises a number of important ethical questions. In our paper, we outline some ethical considerations that need to be addressed in any deployment of this kind of approach as part of a multidimensional public health response. We also, briefly, explore the implications for its use in future infectious disease outbreaks.

## Introduction

### Learning from China

As we write this paper, Europe is at the epicentre of the COVID-19 pandemic. The pandemic has its origins in the emergence, late in 2019, of a novel coronavirus in the Chinese city of Wuhan, which has a population of around 11 million. It is estimated that between the official confirmation of the outbreak and the imposition of a lockdown, around 5 million people left the city. The vast majority went to other parts of China.^1^ The epidemiological implication of this is that the Chinese population outside Wuhan came into contact with many more people infected with COVID-19 than did the world outside China. Despite this, as of 14 April 2020, around 5 months later, China’s total number of cases is 83 306, and its daily case rate is close to zero. By contrast, the global total of cases is now approaching 2 million and doubling every few days in many places.^2^ Compared with other countries, China has been very successful at controlling the spread of COVID-19.^1^

There are a number of features of China’s response to COVID-19 that would be unlikely to be effective or acceptable in other countries. This does not mean that there are not important lessons to learn from China’s success. One element of the approach adopted by China and by several other countries in East and South East Asia that has been highly successful in reducing cases is the use of mobile phone data combined with intensive testing programmes. There is evidence to suggest that the use of this kind of approach might be successfully transferable to other settings with different political and cultural systems.^3^ ^2^

Effective, rapid contact tracing is the cornerstone of effective public health response in the face of infectious disease outbreaks. Its success depends on identifying cases (usually people with symptoms) quickly, gathering information from them about recent contacts and following up and quarantining those contacts to interrupt further transmission of the disease. COVID-19 presents a problem for contact tracing as usually practiced because around 50% of transmissions happen early in infection, before symptoms start, and before test results can be acted on. This means that COVID-19 moves too quickly through the population to be amenable to standard contact tracing methods. The use of a mobile phone app that captures ‘proximity events’—events in which two mobile phones have been close enough for sufficient time for the risk of infection to be high—offers the potential for instantaneous contact tracing from the moment the infection is confirmed.^3^ This has the potential to stop the pandemic.

The modelling for the use of a mobile phone app in COVID-19 and a more detailed description of how this might work have been published elsewhere.^3^ A number of different approaches are currently under development by health systems in many countries around the world. In this paper, our aim is to set out a number of ethical considerations relevant to the use of mobile phone apps to enable rapid contact tracing. These issues will emerge in different ways in different settings.

## Ethical questions

### Benefits and harms

Any consideration of the ethical questions arising in the context of the COVID-19 pandemic has to place great importance on the moral significance of its international spread and the massive scale of its impact. As of 14 April 2020, there have been 1 942 360 confirmed cases and 121 897 deaths globally. These figures are likely to be significant underestimates. It is important to highlight the fact that in addition to those who have died very much larger numbers of people will be suffering symptoms sufficiently serious to warrant hospitalisation and intensive care. In low-income and middle-income countries in which health systems will often not have these facilities, the impact will be much greater.^4^ We are far from the end of the COVID-19 pandemic: these numbers will continue to rise for quite some time.

It hardly needs saying that the saving of lives and reduction of suffering are of immense moral importance and there are strong reasons to support efforts to achieve this. The ethical assessment of an innovation capable of making a contribution to addressing these harms needs to be understood and analysed against the dramatic scale of the deaths and suffering represented by these data.

### Intelligent and unintelligent social distancing

The policy decisions made by governments around the world in response to COVID-19 have been inevitably varied. What is possible, what is required and what is socially and culturally appropriate will differ across the globe. Such differences notwithstanding, many countries have introduced significant restrictions on freedom of movement with disruption to everyday life. One-third of the world’s population is currently living under ‘lockdown’. The terms and enforcement of this vary but all are causing serious economic and other harms to both individuals and institutions with long-term impact. Their impact will be enduring. Many people now and in the future will experience significant suffering as a consequence of these measures.

In the context of public health emergencies, actions are often justified that would not be appropriate outside of such contexts. Such actions do nonetheless require an explicit justification: the mere existence of an emergency does not in itself legitimise any intrusion on the autonomy or privacy of individuals or groups. The justification most commonly offered for the current imposition of lockdowns and other restrictions of movement has been that they are necessary to ensure sufficient ‘physical distancing’ to disrupt the transmission of the infection sufficiently to enable health systems to cope with predicted demand. It is estimated that the overwhelming of health systems, were it to happen, would be one of the main causes of death.

Current approaches to lockdown are, however, blunt tools applied at a national level. They apply to everyone, whether or not they are at risk, affected or immune. This is justified insofar as there is insufficient accurate, reliable information about the risk status of individuals or specific locations, which would enable more finely-tuned decisions to be made reliably. The justification of blanket lockdowns would be weaker were it possible to manage physical distancing in a more evidence-based, risk-adjusted way.^5^ Were this so, it would remain the case that limiting the movements of those people who presented a high risk would be justified. It would not, however, be justified to restrict the movements of those individuals (and possibly populations) who were reliably known not to be contributing to this risk. Rapid contact tracing enabled by the mobile phone app described above—combined with accurate testing—has the potential to be a tool of this kind. The evidence suggests the app has the potential to enable some (likely many) people to return more quickly to their lives. This evidence puts pressure on justifications for blanket lockdowns. The harms presented by such lockdowns also provide support for an argument that the development and implementation of the app as part of a broader package of public health interventions is not only ethically acceptable but also—where feasible—obligatory.^4^ The app is preferable to blanket lockdowns because intelligent physical distancing constitutes the minimum imposition compatible with addressing the epidemic safely.

A fuller analysis would require the relative benefits and harms of other mooted options for non-pharmaceutical intervention to be compared and considered. Controlled or delayed spread of SARS-COV-2 with the primary intention of mitigating against overburdened healthcare resources, herd-immunity by controlled infection in the population, and cyclical lockdowns, have all been considered. Mathematical modelling can be used to compare the likely reductions on the morbidity and mortality, alongside any societal costs of quarantine, mediated by each intervention, Of note, of the options under consideration, however, only contact tracing aims to prevent transmission while explicitly minmising numbers of people in quarantine.^5^

### Privacy

Before the pandemic, questions about data protection, security and privacy were at or close to the top of lists of ethical concerns for many people. Against that background, the use of a mobile phone app built on the gathering and sharing of proximity information, even if pseudonymised, may be seen as deeply concerning, particularly in combination with other socially restrictive measures. Two important questions requiring clarification in this regard are: what is the nature of the infringement of privacy, if there is one, and, can this be justified in the context of the COVID-19 pandemic?

Starting with the question of justification, it seems clear now that some privacy infringements are potentially justifiable where they have the potential to contribute to the saving of many lives and reducing enormous suffering. Imagine a scale running from 0 to 100. At the 0 end of the scale would be someone (Person A) for whom privacy is the concern that trumps all others. People at this end of the scale would place privacy above all other concerns and would be unwilling to give up any privacy to achieve another goal, no matter how important. A person at the other end of the scale would be someone (Person Z) who has no interest at all in privacy and would willingly give up 100% of their privacy for any reason. Person A’s view is likely to be a minority position with regard to this pandemic. The scale of the suffering caused by the COVID-19 pandemic means that if a case can be made that some degree of privacy infringement will save significant numbers of lives and reduce suffering, the intervention may be justified. Any such justification will depend on a clear case being made that the privacy infringement is either necessary or that it is significantly more effective than the alternatives. One aspect of a convincing attempt at justification might be the claim that the privacy infringement is less intrusive than blanket population level lockdowns for everyone. It would, however, also require a convincing case to be made that (i) any privacy impact would be minimised, (ii) that high standards of data security, protection and oversight would be in place, (iii) that there would be transparency about proposed and actual data uses, and (iv) that these would be complemented by other protections, for example, around non-discrimination. This is a useful reminder that Person Z’s is also an ethically problematic position. Of course, an important concern for many people will not only be about their privacy today during the epidemic, but also about their future privacy: will full privacy protections be reinstated after the epidemic? Will data gathered now be used in unacceptable ways later? This final point highlights, importantly, the fact that any justification of infringements of privacy will need to include a convincing account of their scope and duration.

### Possible conflicts between liberty and privacy

The discussion above suggests not only that some constraints on liberty and on privacy may be justified in the context of a global health emergency. It also implies that there may be a tension or trade-off between them. Would it, for example, be ethically justified to retain/impose a blanket lockdown on society as a whole - including those not at risk themselves or a risk to others - *on privacy grounds alone*? Much would depend on the details of the scope of the privacy infringement. However, its potential use in enabling many people now, and ultimately all, to emerge safely from a damaging lockdown provides a strong autonomy-based prima facie argument in favour of the introduction and use of the app even if it were considered to constitute a privacy infringement. It is worth noting that there are at least two ways in which the use of the app has the potential to be autonomy enhancing. The first is its potential to enable people to go about their lives freely without the constraints imposed by a lockdown. The second is that it would provide a tool to enable individual people to make informed choices about how to behave in a socially responsible way e.g. to self-isolate as necessary to reduce the risks to others.

### Should the app be compulsory?

The ideal situation would be for the downloading of the app to be voluntary and for the scale of voluntary uptake to be significant. This is a possibility given that it is believed that an uptake of below 50% would still - in combination with other measures - be sufficient to make an important impact. There are a number of reasons why those who have smartphones will have a strong incentive to sign up. The first of these is that this would ultimately mean that they and everyone else will emerge from the lockdown more quickly and safely. A second is that, by so doing, they will then be enabled to contribute to saving the lives of others, particularly the vulnerable, and those in caring roles, both locally and globally. Appeals to a sense of ‘we are all in this together’ of ‘solidarity’ may be effective.^6^ A third, is related to the impact on the user’s own level of risk. Although primarily aimed at population level impacts, if a person downloads the app - and so do their close contacts - their personal risk will be very significantly reduced. This is because the de facto effects of app uptake will mostly act very locally except in busy urban environments such as the London underground.

What if this does not work? If actual or predicted uptake is insufficient, is there an argument for the use of incentives? Against this background of the scale of the current lockdown, the use of incentives to minimise the length of lockdown while also saving lives might be justified if uptake was insufficient and that there was evidence that greater uptake would release large numbers of people from an avoidable lockdown. The nature of these incentives would need careful consideration on a case-by-case basis. Some possible examples might include: a donation to a nomiated charity, or free mobile phone credit. The use of incentives inevitably raises a number of equity questions with regard to those who do not have access to suitable smartphones and would not have access to these benefits through this route. These would need to be acknowledged and addressed in any defensible policy.^7^

### The responsibilities of institutions and professionals

Thus far, we have been considering ethical questions relating to the use of the app by individuals. There are, however, implications for institutions and professions such as those who manage care homes or places where large numbers of people congregate such as cafes and restaurants. As we emerge from the epidemic into a world in which infection rates are lower but in which there is not as yet a vaccine—a world in which the transmission of COVID-19 needs to be minimised—such people might reasonably be expected to ensure that the level of risk in their establishment or workplace is minimised. In this transition period, people in these positions might reasonably be seen to have an obligation to allow entry only to people who are able to show they are low risk. It might reasonably be judged irresponsible of such an institution to subject residents or customers to avoidable levels of personal risk and to fail to contribute to the suppression of infection transmission in the public interest.^8^

This perspective suggests additional reasons for thinking that the uptake of the app might be high because there is good reason to assume that most people would want to be able to both emerge from the lockdown and also to know that when they went to work or to a café they would be safe to do so, and contributing to the safety of others. This might provide a way for professionals and institutions to meet their obligations and an additional incentive to individuals to act responsibly.

### The ethics of managing emergence from lockdown

If the app can be shown to offer the potential to provide information to enable individuals and those who manage institutions to ensure an intelligent and safe emergence from lockdown, there are good reasons for its use. The ‘if’ here is important, however, because the app’s success will depend not only on the effectiveness of the app itself but also upon the existence of complementary infrastructure such as easy access to reliable testing, support to make sustained self-isolation possible and employment protections to ensure that those who do self-isolate are protected. This suggests the need for an in-depth ethical analysis of the process of emerging from lockdown, potentially into a series of periodic lockdowns with significant impact on the lives and well-being of many people.

### Should the data be deleted at the end of the epidemic?

One way of increasing the chances that people will be willing to download the app and allow it to gather data of proximity events might be for clear legally enforceable commitments to be provided that when the epidemic is over (according to some agreed criteria) the app and its data will be deleted. If this is essential to create the conditions for sufficient uptake and hence for saving lives and reducing suffering, it should be considered. It is not an ethically unproblematic course of action, however. One of the most striking and disturbing aspects of the current pandemic has been the way it has revealed how poorly prepared the world and individual countries are for such an eventuality both in terms of health system resilience, availability of equipment and tests and in terms of reliable epidemiological modelling. Against this background, it is clear that we have responsibilities not only to those who are currently suffering from COVID-19 but also to future generations. If the app is adopted as an intervention, the data it produces could be an invaluable resource for the protection of future generations from serious harm i.e. through research, the development of modelling methods and evaluation of the range of current responses. If these data re to be retained for such uses, a number of important questions about security, oversight, and ownership will need clear and enforceable answers.

### Well-founded public trust and confidence

The successful and appropriate use of mobile phone apps to facilitate instantaneous contact tracing in the context of COVID-19 in democratic countries depends on the establishment of sustained and well-founded public trust and confidence. This applies to the use of the app itself and of the data. The use of ‘well-founded’ here is intended to emphasise that mere presence of trust is insufficient in itself: such trust must be genuinely warranted. The requirements for well-founded trust will vary from country to country and perhaps even from person to person. However, in democratic contexts, in addition to the provision of clearly articulated and justified answers to the questions set out above, requirements are likely to include: the establishment of effective, transparent, accountable and inclusive oversight—perhaps by an ethics oversight body including members of the public; the agreement and publication at the outset of ethical principles by which the use of the intervention will be guided; the use of a transparent, auditable and easily explained algorithm; the highest possible standards of data security; and effective protections around the ownership uses of data.

### Equity, fairness and justice

All public health emergencies and the actions taken to deal with them raise important justice questions because they are situations in which infringements of justice, discrimination and stigma commonly occur. It is also well established that the development and introduction of new technologies are capable of creating new forms of discrimination and further enhancing those that pre-existed the innovation. These can take the form of bias within the technology itself (perhaps because of biased data), biases arising out of the uses to which the technology is put and bias out of the fact that it may be available to some but not all.^6 7^ The response to COVID-19 has been no different to previous public health emergencies in this regard.^8^ Against this background, an important requirement for the credibility of any attempt to justify the use of the mobile phone app as part of a wider set of public health interventions to address the threat of COVID-19 will be recognition of the importance of engaging seriously with equity and justice issues. Notwithstanding the impossibility of addressing all structural issues in the compressed timescale of a pandemic, evidence is needed of a clear, actionable and ambitious plan for addressing these issues.

### Consistency and case comparison

Once the current pandemic is over, there will inevitably be reviews of scientific, epidemiological and medical evidence about which interventions were or were not effective. If it turns out to be the case that the use of instantaneous contact tracing combined with widespread testing is effective, questions will arise about the ethical implications for its use in other infectious disease outbreaks. Would it, for example, be acceptable or even required for a specifically designed app to be used each year in the context of seasonal influenza? These are important ethical questions. Although there are differences, there are also morally significant similarities between COVID-19 and seasonal influenza. For example, while its transmission rate is generally lower than COVID-19, the numbers of deaths internationally from seasonal influenza are very large indeed.^9^ One important difference, at present, between the two diseases is that mechanisms capable of developing a vaccine each year with some degree of effectiveness against seasonal influenza are in place. This may suggest that, unless judged less harmful or more effective than vaccination, the use of the app in seasonal influenza may not be justified. However, it is possible that apps will be appropriate in other settings and, where likely to be effective, constitute an important and ethically justified part of the public health toolkit.

## Conclusion

In this paper, we have set out a number of pressing ethical questions raised by the proposed use of a mobile phone app, the collection of proximity data for the control of the COVID-19 pandemic, and the safe emergence of populations from government-imposed lockdowns. Scientific and epidemiological evidence suggest that an app of this kind has the potential to contribute to reducing the suffering caused by the pandemic and minimise the harms caused by long periods of lockdown. These benefits and the avoidance of harms are clearly of great moral significance. If they are to be realised, however, several other ethical requirements need to be met. We have highlighted a number of such requirements which deserve attention in any ethically justified use of this technological intervention. In the UK, there is early empirical evidence that a high proportion of the population would choose to download the app under current circumstances, given adequate protections. In an on-line survey of predicted user-acceptance conducted by our collaborators, 74% of respondents said they would definitely or probably install a contact-tracing app.^10^ Before they are invited to do so, they need to be assured that adequate protections and oversight are in place. A profoundly important ethical question presented by this technology concerns the problem of how and whether societies can find ways to benefit from the potential of algorithmic approaches to improve public and individual health, while also ensuring that the legacy of the deployment of these technologies does not impact negatively on future generations.

## Footnotes

There is debate about the accuracy of the figures coming out of China but broad agreement about the success of their intervention in reducing the number of infections.

The effectiveness and reach of any implementation of the app in democratic societies will inevitably be affected by varying configurations of state-citizen relationships, as well as by the roles of civil society groups and non-governmental actors.

The question of what constitutes adequate information about infection status for a population effect may be answered differently by different systems, ranging from self-reported symptoms through to clinically validated test results.

Subject to a number of caveats discussed later in the paper.

The true effectiveness and sustainability of these interventions remains to be seen; benefits and harms of interventions should be evaluated post-implementation and alternative strategies (including combinations of approaches) continuously reconsidered.

It is important to note that there are circumstances in which, and many people for whom, appeals to solidarity might also be exclusionary or deepening of existing social divisions.

These and other equity questions are expanded on below.

This paragraph needs to be understood in the context of those made in the later section on ‘Equity, fairness, and justice’.
